# Improving psychosocial well-being and parenting practices among refugees in Uganda: Results of the journey of life effectiveness trial – CORRIGENDUM

**DOI:** 10.1017/gmh.2024.51

**Published:** 2024-05-03

**Authors:** Lindsay Stark, Melissa Meinhart, Sabrina Hermosilla, Rehema Kajungu, Flora Cohen, Gary S. Agaba, Grace Obalim, Justin Knox, Patrick Onyango Mangen

Upon publication of this article the 2nd affiliation for author Flora Cohen, University of Illinois Urbana Champaign, was omitted. The full affiliation list for Flora Cohen, should be:

Brown School of Social Work, Washington University in St. Louis, St. Louis, MO, USA

University of Illinois Urbana Champaign, IL, USA.

The article has been updated to include the missing information.
